# *Alistipes senegalensis* is Critically Involved in Gut Barrier Repair Mediated by *Panax* Ginseng Neutral Polysaccharides in Aged Mice

**DOI:** 10.1002/advs.202416427

**Published:** 2025-07-03

**Authors:** Dandan Wang, Hui Wang, Yingna Li, Jing Lu, Xiaolei Tang, Dan Yang, Manying Wang, Daqing Zhao, Fangbing Liu, Shuai Zhang, Liwei Sun

**Affiliations:** ^1^ Research Center of Traditional Chinese Medicine the Affiliated Hospital to Changchun University of Chinese Medicine Changchun 130000 China; ^2^ Northeast Asian Institute of Traditional Chinese Medicine Changchun University of Chinese Medicine Changchun 130000 China

**Keywords:** *Alistipes senegalensis*, gut barrier, indole, neutral polysaccharides, Panax ginseng

## Abstract

Ginseng polysaccharides (GPs) are known to have beneficial effects on the gut epithelium and age‐related systemic‐inflammation through regulation of gut microbiota. However, the underlying pathways and key members of the microbial community involved in this process are poorly understood. In this study, administration of ginseng neutral polysaccharide (GPN) is found to alleviate gut leak and low‐grade inflammation, concomitantly with improving the physiological function aged mice. Fecal microbiota transplantation and fecal conditioned medium are used to assess the specific involvement of gut bacterial metabolites in the effects of GPNs. Comprehensive multi‐omics analyses showed that GPN significantly enriched the abundance of *Alistipes senegalensis*, an indole‐producing commensal bacterium. Increased expression of tight junction‐associated proteins, as well as activation of gut stem cells, are found to be mediated by the AhR pathway, indicating the causal mechanism by which GPN reduced increases in gut permeability. The results are verified in Caco‐2/THP‐1 cells, *Caenorhabditis elegans*, and enteroids. To the knowledge, this is the first identification of an integral functional axis through which GPN and functional metabolites of *A. senegalensis* influence the gut barrier and reduce systemic inflammation, providing clues for the potential development of innovative plant polysaccharide treatment strategies to promote healthy aging.

## Graphical abstract

An integral functional axis through which GPN and functional metabolites of *Alistipes senegalensis* can hydrolyze tryptophan into indoles. Furthermore, the *Alistipes senegalensis*‐indole‐AhR pathway is found to be associated with increased expression of tight junction‐associated proteins and activation of gut stem cells. Through these mechanisms, GPN alleviates systemic inflammation and mitigates the accelerated‐aging phenotype.

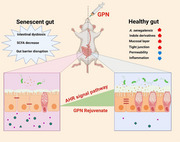

## 1 Introduction

Aging is a complicated physical process accompanied by cellular senescence, autophagy dysfunction, and dysbiosis of the gut microbiota. In particular, the presence of low‐grade inflammation is widely recognized as an important driver of metabolic syndrome,^[^ ^1^, ^2^ ^]^ Alzheimer's disease, and other age‐related diseases, adversely affecting the quality of life and even reducing the lifespan of older adults.^[^ ^3^ ^]^ Although the exact etiology of the increased inflammation seen with aging remains elusive, current evidence suggests the involvement of increased “gut leakiness” and “inflammaging”, both of which exacerbate the senescence process.^[^ ^4^, ^5^ ^]^ Specifically, increased gut permeability triggers the transfer of endotoxins and antigens from the intestine to the circulation, and the resultant low‐grade inflammation can also lead to further damage of the intestinal epithelial barrier.^[^ ^6^ ^]^

The epithelial cells of the intestinal mucosa are organized in a single layer that forms a highly selective barrier. The integrity of this barrier is dependent on inter‐cellular junctional complexes formed by the proteins zona occludens‐1 (ZO‐1), occludins, and claudins. Research has shown that knockout of ZO‐1 increased gut permeability and the influx of solute molecules in the intestinal epithelial cell line Caco‐2, indicating the importance of this protein in the maintenance of gut barrier function.^[^ ^7^, ^8^ ^]^ In recent years, evidence of how the gut barrier alters with aging, in both humans and laboratory animals, has emerged. Due to the rapid turnover rate of the intestinal epithelium, maintenance of its integrity is closely linked to the self‐renewal of intestinal stem cells (ISCs) during the lifespan. ISCs are located in a specialized region of the intestinal crypt, and aging is accompanied by ISC dysfunction and exhaustion, which might represent a key mechanism underlying age‐associated increases in intestinal permeability and systemic inflammation.^[^ ^9^ ^]^

The gut microbiota has generated considerable interest for its multiple roles in host metabolism and overall functioning.^[^ ^4^, ^10^ ^]^ An important feature of microbial dysbiosis during aging is the overgrowth and colonization of endotoxin‐producing pathobionts followed by the loss of protective commensals,^[^ ^11^, ^12^, ^13^ ^]^ including genera *Bacteroides, Alistipes, Parabacteroides, Faecalibacterium, Ruminococcus, Coprococcus, Roseburia, Coprobacillus, Lactobacillus, Bifidobacterium*, and *Akkermansia*.^[^ ^14^ ^]^ The systemic effects of the gut microbiota are attributed mainly to microbiota‐derived metabolites, which are in close proximity to the gut epithelium and orchestrate the differentiation of stem cells and the integrity of the intestinal barrier.^[^ ^15^ ^]^ Patients with ulcerative colitis (UC) show a lower abundance of *Firmicutes* and *Lachnospiraceae*, associated with reduced levels of short‐chain fatty acids, increased intestinal permeability, and gut inflammation.^[^ ^16^ ^]^ Urolithin A,^[^ ^17^ ^]^ derived from polyphenolic compounds in berries, has been found to alleviate gut barrier dysfunction in addition to anti‐inflammatory and anti‐aging activities. Indoles and kynuric acid have also been found to influence the aging process and activate gut stem cells.^[^ ^15^ ^]^ However, little is known of the complicated reciprocal regulation between microbiota metabolites, the gut barrier, and aging. Therefore, the development of effective strategies targeted toward restoring the intestinal epithelial barrier is a much‐needed step to counteract inflammaging and age‐related diseases that adversely affect the health and well‐being of older adults.

*Panax ginseng* C. A. Meyer is widely used as a Chinese medicine and functional food throughout the world. It contains a variety of active compounds, including ginsenosides,^[^ ^18^ ^]^ essential oils, peptidoglycans, and phenolic compounds, that have been demonstrated to be responsible for its immunomodulatory^[^ ^19^ ^]^ and anti‐aging properties.^[^ ^20^ ^]^ Notably, ginseng polysaccharides (GPs), a potential herbal prebiotic, have been found to improve intestinal metabolism and modulate the abundance of beneficial commensals (particularly enhancing the growth of *Lactobacillus spp*. and *Bacteroides spp*.) in the gut microbiota.^[^ ^21^ ^]^ Our previous study showed that GPs could protect the gut epithelium by alleviating autophagic dysfunction and reducing the contents of bacteria producing high levels of lipopolysaccharide (LPS). The precise mechanism, however, has not been investigated in depth. In the present study, the effects of GPs in combating gut leakage through increasing the abundance of *Alistipes senegalensis* and its associated indole production were investigated in aged mice. To the best of our knowledge, this is the first identification of an integral functional axis consisting of GPs, *Alistipes senegalensis* metabolites, and the gut barrier to alleviate the effects of aging.

## 2 Results

### 2.1 GPN Isolation and Physicochemical Characterization

**Figure 1:**
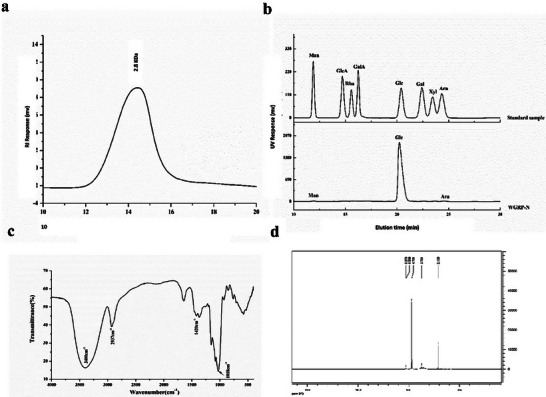
Structural characterization of polysaccharides. a) Elution curve of WGP‐N on HPGPC column; b) Monosaccharide composition analysis of WGP‐N; c) FT‐IR spectrum of WGP‐N; d) ^13^H‐NMR spectra of WGP‐N.

The yield of crude polysaccharide extracted from ginseng was ≈9.8%. After purification using a DEAE cellulose column, two fractions were isolated. Ginseng neutral polysaccharide (GPN) was obtained with an 88% yield and ginseng acidic polysaccharide (GPA) was obtained with a 12% yield; subsequent experiments were conducted using the high‐yield GPN. GPN exhibited a single peak on the HPGPC spectrum, indicating a homogeneous fraction with a MW of 2.80 kDa (**Figure** **1**a). GPN was found to contain mainly glucose (Glc, 99.2%), mannose (Man, 0.4%), and arabinose (Ara, 0.4%), as shown in Figure 1b. The FT‐IR spectra were analyzed (Figure 1c), with the strong bands in the region of of 3400 cm^−1^ attributed to the O‐H stretching vibrations of hydrogen bonds and free hydroxyl groups. The band in the region of 2937 cm^−1^ represented stretching vibrations of C‐H in the sugar groups. The angular vibration absorption peak of C‐H at 1420 cm^−1^ and the vibration absorption peak of C‐O‐C ≈1018 cm^−1^ indicated the presence of pyran ring conformations. The ^1H^NMR spectra of GPN (Figure 1d) showed that δH 5.28 represented the →4)‐α‐D‐Glcp‐(1→ glycosidic bond and δH 4.70 is the α‐D‐Glcp‐(1→ linkage, in addition to multiple absorption peaks between δH 3–4.^[^ ^22^ ^]^ It can thus be seen that GPN is a water‐soluble neutral glucan of ginseng.

### 2.2 GPN Treatment Alleviated Senescence Phenotypes and Suppressed Systemic Inflammation via Restoration of the Gut Barrier in Older Mice

**Figure 2:**
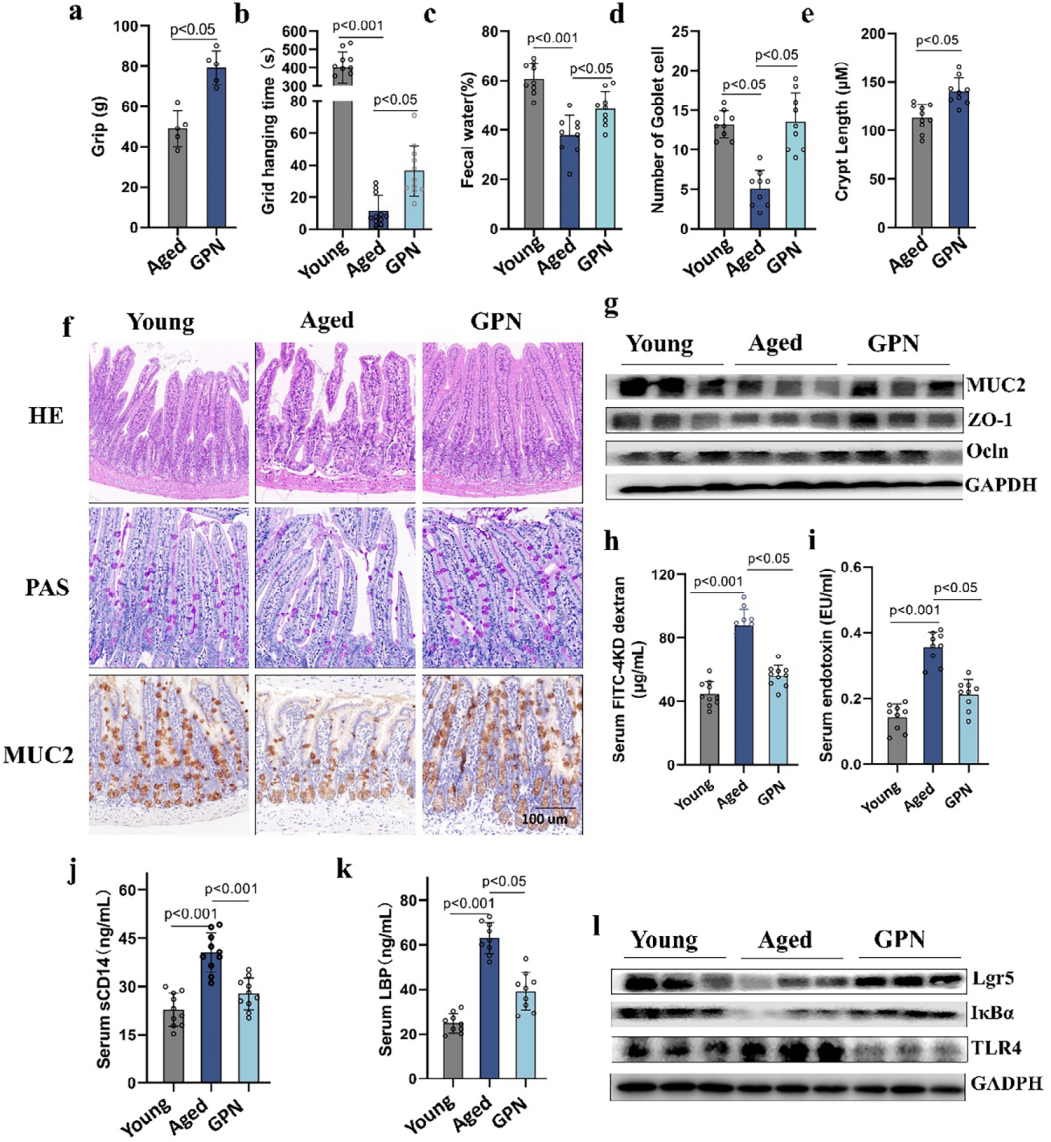
GPN treatment effectively alleviates the aging phenotype and systemic inflammation by restoring the intestinal barrier in aged mice. Female aged BalB/c mice (72‐week‐old) were treated with or without GPN for 8 weeks, young mice were considered as control (n = 10). a) Grip strength of contralateral forelimb. b) Grip hanging time. c) The diameter water content of feces. d) Number of PAS^+^ cells each villus. e) Measurement of small intestinal crypt length. f) The morphology images of small intestinal sections with H&E staining, PAS staining and Muc2 IHC (dark brown), Scale bar = 100 µm. g) The protein expression of tight junction (ZO‐1, Occludin) and mucus synthesized by goblet cell (Muc2) in small intestine tissue. h) Systemic leaky gut marker levels in serum including FITC 4kDa‐dextran i) Endotoxin j) Serum soluble CD14 k) as well as LPS binding protein (LBP). (l) The protein expression of ISC activity (Lgr5) and inflammation‐related proteins (IκBα and TLR4) in small intestine tissue. Data are performed as the mean ± s.e.m.

It was found that GPN supplementation markedly improved the overall health and physiological function and reduced systemic inflammation after 8 fs of administration to 18‐month‐old mice. As shown in **Figure** **2**a,b, the grip and hanging time of the older mice were markedly increased after GPN treatment. As the intestine aged, the chemical defense barrier was impaired, as seen in the presence of fewer secretory goblet cells, and the prevalence of astriction was increased, likely due to reductions in fecal water. Treatment with GPN increased the number of goblet cells per villus and the fecal water by 180% and 19%, respectively, compared to the untreated aging group without affecting food and water intake (Figure 2c,d). The morphological data (Figure 2f) indicated reduced structural integrity of the small intestine, and the intestinal villi were shorter and partly shed in the 18‐month‐old mice. In addition, the intestinal mucosa was thinner than that in the 2‐month‐old controls, and the connective tissue of the proper layer was loosened. After GPN treatment, these morphological changes were alleviated to a significant extent. The regenerative potential of the intestine was evaluated by the crypt depth. As shown in Figure 2e,g, treatment with GPN increased both crypt depth and villus length and restored the levels of the tight‐junction proteins ZO‐1, Occludin, and MUC2, the expression levels of all proteins were quantified in Figure S5a (Supporting Information).

The degree of systemic inflammation potentially caused by gut leakage. As expected, the levels of the intestinal stem cell marker Lgr5 were significantly reduced in older mice but were restored to control levels after GPN intervention (Figure 2l; Figure S5b, Supporting Information). The impairment of the intestinal epithelium was evidenced by the increased levels of LPS‐binding protein (LBP), endotoxin, sCD14, and diffusion of FITC‐dextran (Figure 2h–k). Due to the increased gut permeability, the influx of antigens into the blood activated pattern recognition receptors and triggered downstream signaling cascades. With the restoration of barrier function in the older mice, the activation of TLR4 and IκBα returned to the level seen in the control group (Figure 2l).

Collectively, these results demonstrated that GPN could enhance health, alleviate systemic inflammation, and mitigate both impairment of the intestinal barrier and stem cell exhaustion in aged mice. However, GPN did not exhibit any beneficial effects in Caco‐2 cells or microbiota‐depleted mice (Figure S1, Supporting Information). Therefore, it is hypothesized that the gut microbiota may mediate the effects of GPN.

### 2.3 Gut Microbiota Remodeling was Indispensable for the Beneficial Therapeutic Effects of GPN

**Figure 3:**
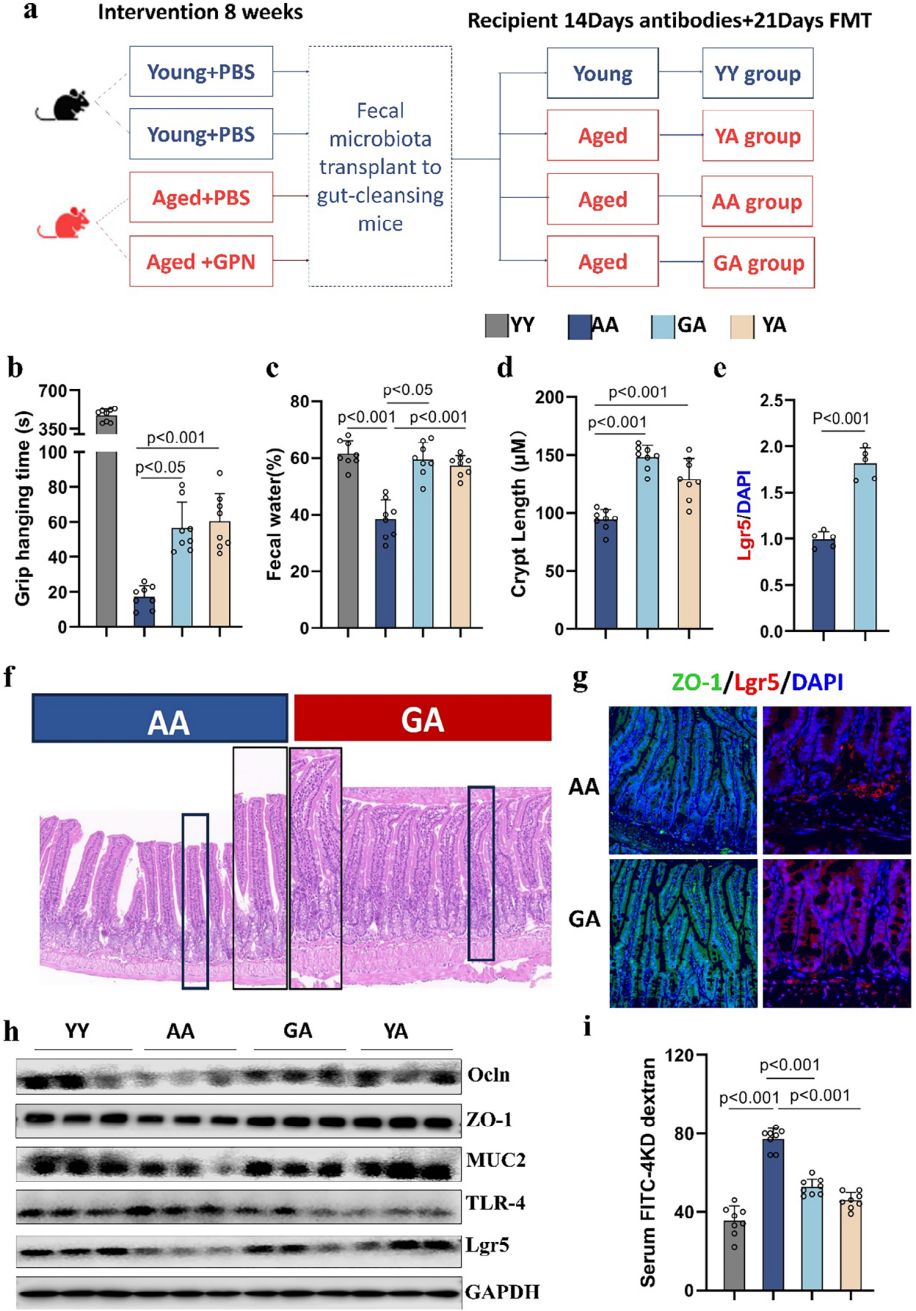
Gut microbiota‐dependent therapy effects of GPN. a) Mouse grouping and microbiota transfer timeline overview. Aging traits measured in recipients after 21 days including b) Grip hanging time, c) Fecal water. d) Crypt length. e) Mean fluorescence intensity of Lgr5 (Fold change). f) Visualized images of H&E staining and g) immunofluorescence staining of ZO‐1 (red), Lgr5 (green), DAPI (blue) in small intestine, Scale bar = 50 µm. h) Immunoblot analysis of tight junction, ISC activity and inflammation proteins in small intestine (n = 6). i) Intestinal permeability. Data were analyzed using one‐way analysis of variance followed by Bonferroni's post hoc test and false discovery rate correction for multiple testing. Data are presented as the mean ± s.e.m.

Despite the demonstration that GPN decreased systemic inflammation, and intestinal permeability, and ameliorated stemness attenuation harbored by aged gut, direct evidence for the participation of the microbiota in this process remained elusive. Thus, the effects of fecal microbiota transplantation (FMT) were investigated. As depicted in **Figure** **3**a and 2‐month‐old and 18‐month‐old donor mice were divided into 4 groups for an 8‐week gavage of GPN or PBS. The groups receiving FMT were treated with a cocktail of antibiotics to deplete the microbiota before receiving the gut microbiota from the donor mice. After colonization for 21 days, the senescent phenotype of the GPN‐recipient aged mice (GA group) was found to be reduced. Specifically, the grip hanging time of the GA mice (Figure 3b) was increased to levels comparable to or higher than that of the YA control group (aged mice that accepted microbiota from young donors). In addition, the amount of fecal water in the GA mice was markedly increased to ≈80% compared to the AA control group (aged mice that accepted microbiota from aged donors) (Figure 3c). Morphologically, the mucosa of the intestines in GA mice was thicker than that of the AA mice, and the crypt depths and villus lengths were significantly restored in the GA group (aged mice that received microbiota from GPN donors) (Figure 3d,f; Figure S2, Supporting Information). In addition, the expression of ZO‐1 and Lgr5 in the older mice was significantly increased after receiving microbiota from GPN‐treated donors (Figure 3e,g). Thus, the systemic inflammation and gut permeability in the older recipients were recovered to a certain extent (Figure 3h,i; Figure S5c, Supporting Information).

These data demonstrated that GPN treatment‐mediated repair of the intestinal barrier occurred indirectly through the bacteria rather than acting directly, and thus the effect of GPN on the intestine is sustained. We then asked whether the effects of GPN were mediated by gut microbiota‐derived metabolites or the structural aspects of the bacteria themselves.

### 2.4 GPN‐Mediated Repair of Age‐Related Gut Permeability was Associated with Bacterial‐Derived Metabolites

To elucidate the complex and ambiguous crosstalk through which the gut microbiota influenced intestinal tight junctions and the aging phenotype in older mice, and to verify whether the therapeutic effected originate from the gut microbiota themselves or from the metabolites produced by intestinal bacteria, a fecal conditioned medium (FCM) containing a broad spectrum of metabolites was prepared.

**Figure 4:**
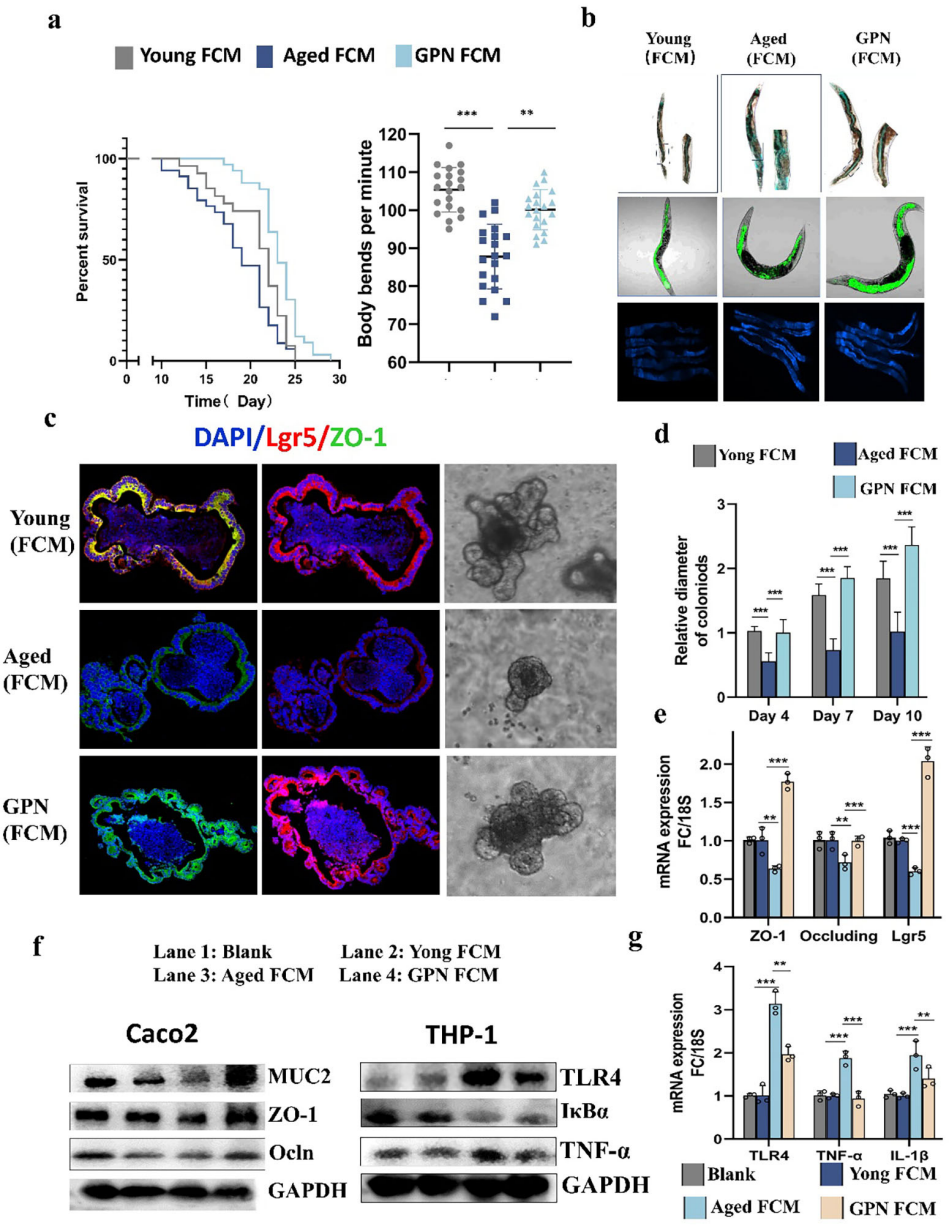
The repairment of GPN on age‐related gut leak are associated with bacterial‐derived metabolites. a) Survival curves and Locomotor performances of WT *C. elegans* strain N2 fed on *E. coli* OP50, 1:1 mixture of *E. coli* OP50 and FCM of aged or GPN, (N = 80 for each group). b) DIC images of N2 animals after soaking in blue food dye for 3 h on Day 13, scale bar = 100 µm (left) and 50 µm (right, showing higher magnifications of boxed regions in the left), representative images of ges‐1::GFP (mit) in SJ4143 worms and autofluorescence of lipofuscin accumulation in N2 nematodes, scale bar = 100 µm. c) Representative images of Lgr5 staining (green), ZO‐1 staining (red) and DAPI staining (blue) in small intestinal organoids from different groups at Day 7 with 3 independent experiments, scale bar = 50 µm. d) Size of small intestinal organoids quantified from different groups on day 7. Treatment of Caco‐2 cell monolayers (cultured with THP1 cells) with FCM, e,g) real‐time PCR and f) Western blot were performed to detect mRNA levels and the protein of tight function (ZO‐1, Occludin), Muc2 and inflammation (TNF‐ɑ, TLR4, IκBɑ). Data are shown as mean ±s.e.m. **p < 0.01 ***p < 0.001.

The nematode *C. elegans* is an excellent model for dissecting the interaction between bacterial metabolites and the host due to its advantages of having signal microbiota and a reproducible lifespan. Feeding the worms with the FCM of the GPN group resulted in a series of anti‐aging benefits including prolonged lifespan, improved mobility (**Figure** **4**a), and better intestinal function, including activation of intestinal mitochondria and reductions in intestinal permeability and Lipofuscin pigmentation (Figure 4b) in *C. elegans*. In addition, the GPN FCM could also improve the regenerative potential and barrier function of ISCs, which were verified by the detection of the diameters of the organoids and expression of Lgr5 and ZO‐1 on day 7 in the primary small intestinal organoids derived from BALB/c mice (Figure 4c,d). Collectively, these results confirmed that bacteria‐derived metabolites derived from GPN‐treated mice, which recapitulated the effects of FMT on intestinal function, could restore the structure of intestinal tight junctions and alleviate the aging phenotype.

Here we attempted to further clarify the relationship between reduced inflammation and increased expression of tight junction proteins. To confirm that the actions of GPN on the intestinal epithelium involved improving tight junctions to reduce inflammation, or vice versa, we used a coculture system of human macrophages (THP‐1‐differentiated; in the lower chamber) and human intestinal epithelial cells (Caco‐2; in the upper chamber). When Caco‐2 cells were exposed to GPN FCM, a significant upregulation of tight junction‐associated mRNAs and proteins was observed in Caco‐2 monolayers compared to the control group. This enhancement of barrier function subsequently led to a reduction in inflammatory factors in macrophages (Figure 4e,g,f; Figure S5d,e, Supporting Information). While cocultured macrophages were exposed to FCM, no significant decrease in inflammation or increase in gut barrier function was observed (data not shown). Overall, these findings indicate that GPN‐related metabolites predominantly enhance the expression of tight junction proteins in intestinal epithelial cells, thereby reducing epithelial permeability and consequently mitigating inflammation, rather than reducing inflammation to repair the intestinal epithelium. In addition, the long‐term effects of GPN‐related metabolites were evaluated in *C. elegans* throughout their lifespan (Figure S3, Supporting Information). Despite a decline in the therapeutic benefits over time after treatment completion, the extension of lifespan remained significant, suggesting sustained efficacy of GPN.

### 2.5 GPN Elevated Tryptophan Metabolism and Indole Derivative Contents, Inducing AhR Activation, Stemness Promotion, and Barrier Restoration

**Figure 5:**
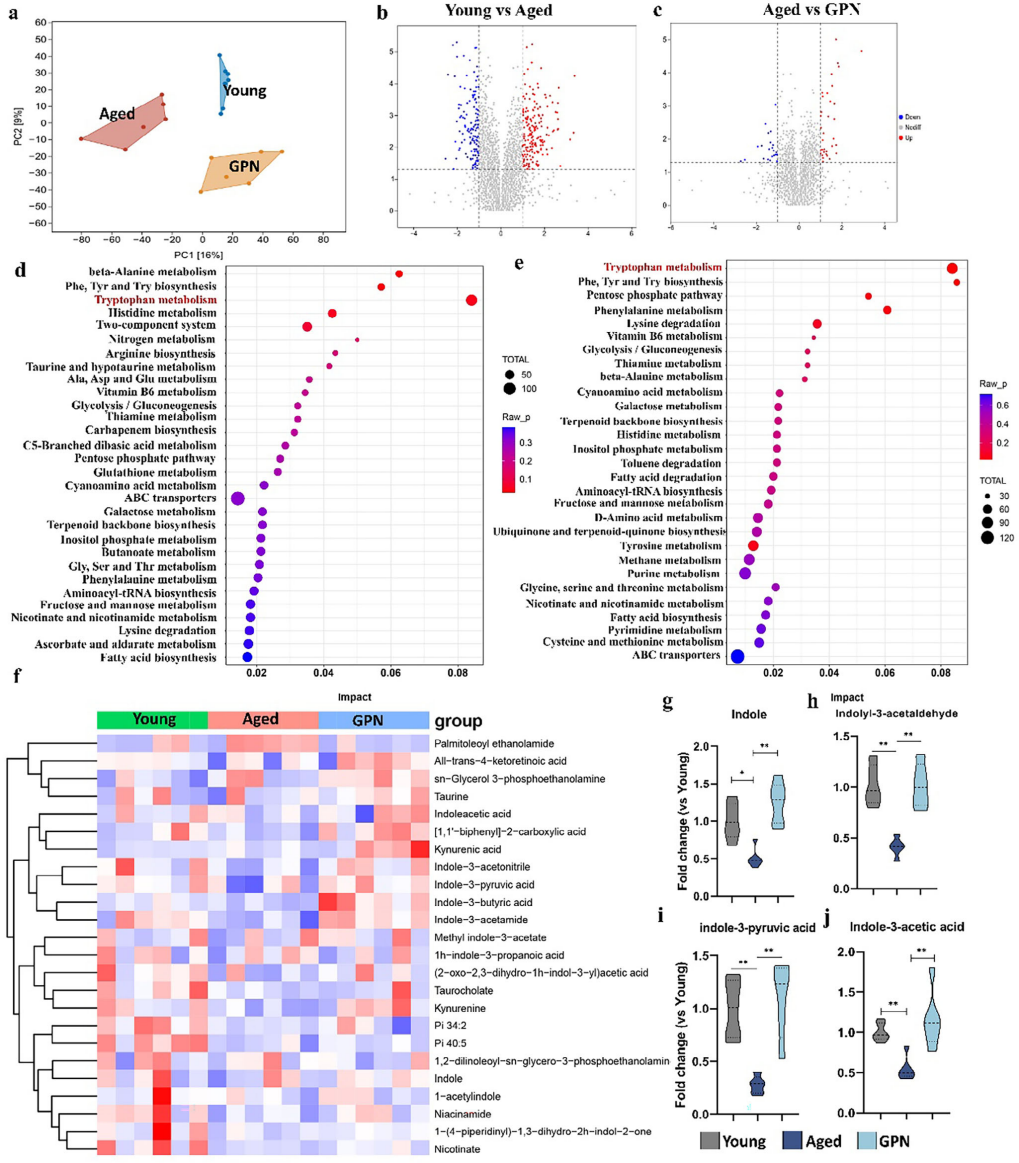
GPN elevates tryptophan metabolism and indole derivative content. a) Principal component analysis (PCA) of metabolomics data measured from the faeces of Young and Aged mice. b,c) The volcano plot for different metabolites between Young versus Aged and Aged versus GPN mice. The enriched KEGG metabolite pathways of d) Young and Aged groups, e) Aged and GPN groups. f) Hierarchical clustering heatmap of metabolites in the feces of Young, Aged and GPN groups g–j) Quantitation for relative abundance of indole and its derivatives.

It was found that GPN enhanced the gut barrier function and decreased inflammation in aged mice through microbiota‐derived metabolites, but the precise pathway remained ambiguous. We thus conducted untargeted global metabolomics and principal component analyses in the feces. It was found that the aged gut was associated with a different metabolite signature relative to that of the young group, and the metabolites from GPN‐fed mice formed a cluster distinct from the aged group (**Figure** **5**a). In addition, the volcano plot (Figure 5b,c) indicated that compared with the Young group, the Aged group exhibited 216 up‐regulated and 151 down‐regulated metabolites. Furthermore, the GPN‐treated mice showed 34 up‐regulated and 22 down‐regulated metabolites relative to the Aged group. Specifically, the unbiased random forest and differential abundance analyses showed that aged mice had reduced levels of tryptophan metabolism (Figure 5d,e). Further, age‐related metabolites, such as taurine, indoles, indole derivatives, and kynuric acid, were higher in the GPN‐treated mice compared with the aged mice (Figure 5f). We subsequently measured the levels of key metabolites in the tryptophan metabolism pathway (Figure 5g–j). The results showed that nicotinate, indole, and its derivatives were reduced in aged mice, whereas GPN intervention restored their levels. It has been reported that indoles and their derivatives were previously shown to be effective in repairing the epithelial barrier and activating Lgr5^+^ stem cells^[^ ^23^ ^]^ through the aryl hydrocarbon receptor (AhR), a transcription factor activated by ligands that integrates microbial and metabolic factors in a ligand‐activated manner.

**Figure 6:**
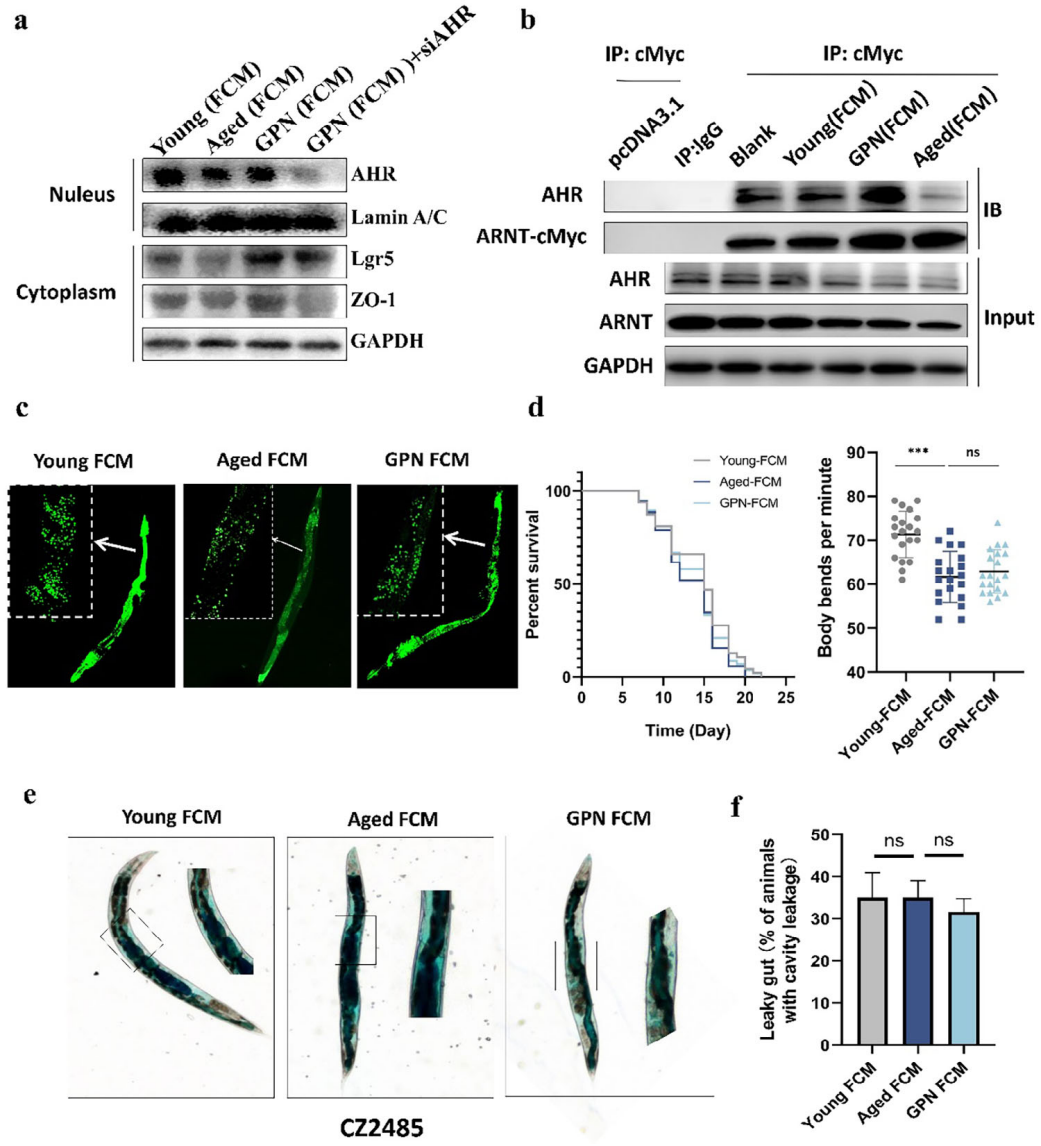
The beneficial effect of GPN involves the AhR/ARNT binding. a) Proteins of Caco‐2 cells pretreated with FCMs were extracted and western blotting was performed to detect the expression of AhR, ZO‐1 and Lgr5. b) Co‐IP and immunoblot analysis for detecting the binding of ARNT and AhR in HEK293T cells. c) Representative images of ahr‐1::GFP in UL1709 worms, n = 15. d) Survival curves (n = 75 each group) and locomotor performances (n = 25 each group) of N2 strain worms. e,f) intestinal permeability (leaky gut) using Smurf assay of CZ2485 strain, n = 15 each group, scale bar = 100 µm, Data are presented as the mean ± s.e.m.

Next, to determine whether the protective effects of GPN were associated with the indole‐AhR pathway, we evaluated the levels of several key AhR‐related proteins in Caco‐2 cells and *C. elegans*. **Figure** **6**a (Figure S5f,g, Supporting Information) shows that metabolites prepared from GPN‐treated mice significantly increased the expression of nuclear AhR, ZO‐1, and Lgr5. As expected, GPN FCM was unable to restore ZO‐1 levels and stem cells when AhR was knocked down. Further, decreased tryptophan metabolism due to aging^[^ ^24^, ^25^ ^]^ reduced the transport and activation of AhR while treatment with GPN FCM markedly enhanced binding between AhR and its transporter protein ARNT in HEK293T cells, as shown in Figure 6b. The increased activation of AhR activation was also confirmed in the UL1709 nematode strain (Figure 6c). Furthermore, we compared the therapeutic effects of GPN FCM with those of indole, a widely accepted AhR ligand. As anticipated, GPN FCM demonstrated comparable efficacy, which further supports the hypothesis that indole and its derivatives may account for the beneficial effects of GPN‐related metabolites (Figure S4, Supporting Information).

The data shown in Figure 4 confirmed a series of beneficial effects of GPN FCM, and to further examine the role of AhR in gut barrier repair, the CZ2485 strain (an AhR^‐/‐^ mutated nematode strain)^[^ ^26^ ^]^ was treated with FCM. The FCM prepared from GPN‐treated aged mice was unable to restore the gut barrier integrity and physiological function in AhR‐1 mutant nematodes (CZ2485), as seen in Figure 6d–f, implying that the microbiota‐tryptophan metabolism‐AhR axis was the crucial pathway for the beneficial effects of GPN.

Collectively, the neutral active polysaccharides isolated from ginseng elevated tryptophan metabolism and indole contents through modulation of the gut microbiota, followed by activation of the AhR pathway to trigger the expression of genes associated with the intestinal barrier and stem cells, together with the suppression of systemic inflammation. Nevertheless, the specific details of the mechanism underlying GPN‐induced epithelial integrity require further investigation.

### 2.6 *Alistipes senegalensis* Contributed Significantly to Indole Accumulation Induced by GPN Treatment

**Figure 7:**
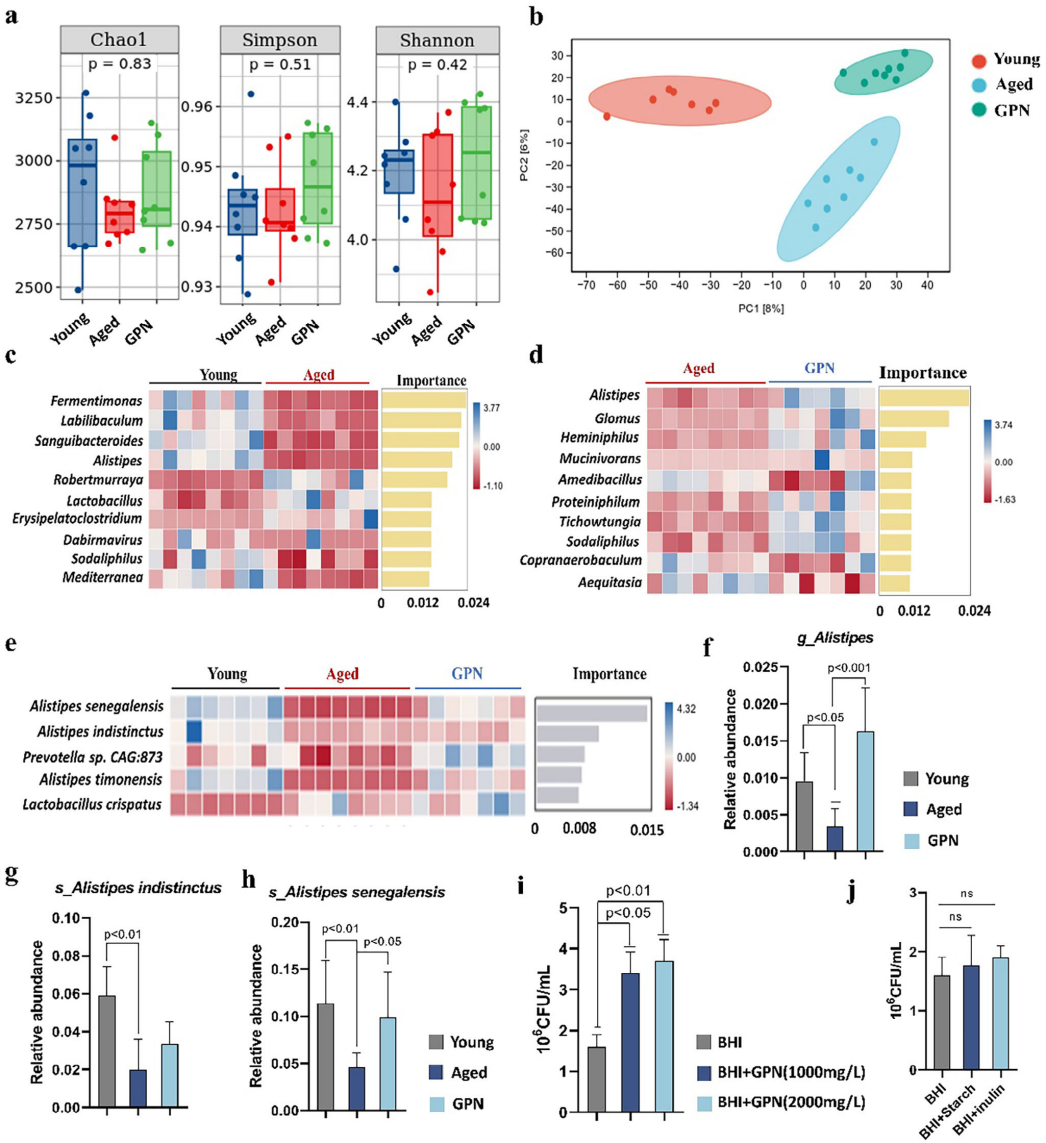
GPN treatment affects the bacterial composition of the gut microbiota. a) Comparison of alpha‐diversity indices (Chao1 index and Shannon index) and beta‐diversity among groups. b) Principal component analysis (PCA) of among groups. R value through ANOSIM analysis, GPN versus Young (R = 0.1153, p = 0.0813); Aged versus Young (R = 0.6076, p = 0.002). Heatmap of average relative abundance of top 10 generas in c) young and aged mice groups and d) aged with and without GPN groups. e) Heatmap of top 5 most abundant bacterial species among groups. f) Relative abundance of *Alistipes* genus from metagenome data. g,h) Relative abundance of *Alistipes indistinctus* species and *Alistipes senegalensis* species. i,j) Colony‐forming units (CFU) of *Alistipes senegalensis* were measured after anaerobic culturing with GPN, inulin and starch. Data are presented as the mean ± s.e.m.

**Figure 8:**
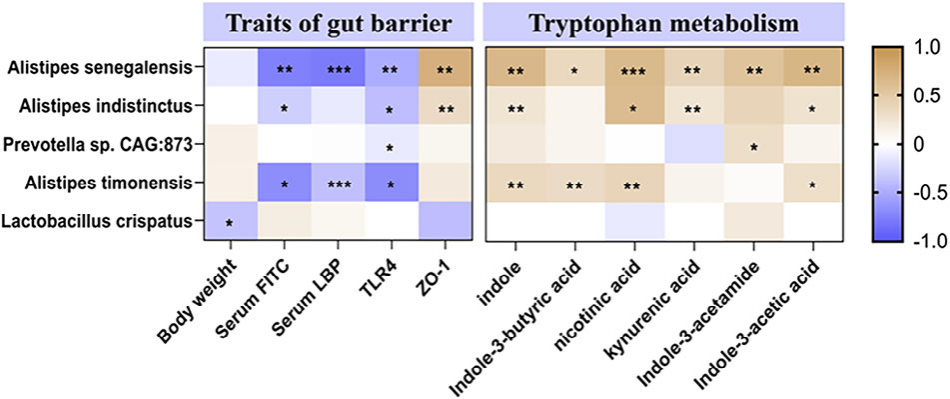
Spearman's correlation analysis between the top 5 bacteria species enriched by GPN and traits of gut barrier and tryptophan metabolism levels.

Next, the specific bacterium responsible for tryptophan metabolism and indole generation was investigated by mining the metagenomic sequencing data. This allowed analysis of the response of the gut microbiota to physiological processes. **Figure** **7**a illustrated the differences in the Chao1, Simpson, and Shannon indices across the groups, although these differences were not statistically significant. The PCA plots (Figure 7b), however, revealed distinct separations, indicating that the composition of the microbiota differed markedly among the young, aged, and GPN‐treated groups. The bacteria that were reduced in the aged mice compared to the young group were then investigated (Figure 7c). The *Alistipes* genus drew our attention because of its significant enrichment in the GPN‐treated group (Figure 7d,f). The identification of the specific bacterium involved is significant in determining the mechanism of GPN‐mediated gut protection. Therefore, the five most enriched bacterial species following GPN supplementation were selected for further analysis, as illustrated in Figure 7e. Among these species, *A. senegalensis* exhibited the highest abundance within the *Alistipes* genus and was significantly enriched by GPN treatment. The quantification of *Alistipes* genus and the top two most enriched species is shown in Figure 7g,h. In addition to the metagenomic analysis in mice, we also assessed whether the growth of *A. senegalensis* was stimulated by GPN in vitro. The results (Figure 7i,j) showed that increases densities of *A. senegalensis* in the BHI plate with GPN after 120 h of culture. The addition of starch and several herbal polysaccharides to the BHI as a control resulted in no significant promotion of *A. senegalensis* growth, consistent with recent research,^[^ ^27^ ^]^ and supporting the specific stimulatory effects of GPN. We next performed a correlation analysis between the bacterial abundance and indole levels, as well as with inflammaging‐related parameters. Among these species, *A. senegalensis* was not only the highest in abundance, but was also positively correlated with the level of tryptophan metabolism and negatively correlated with systemic inflammation in aged mice (**Figure** **8**).

**Figure 9:**
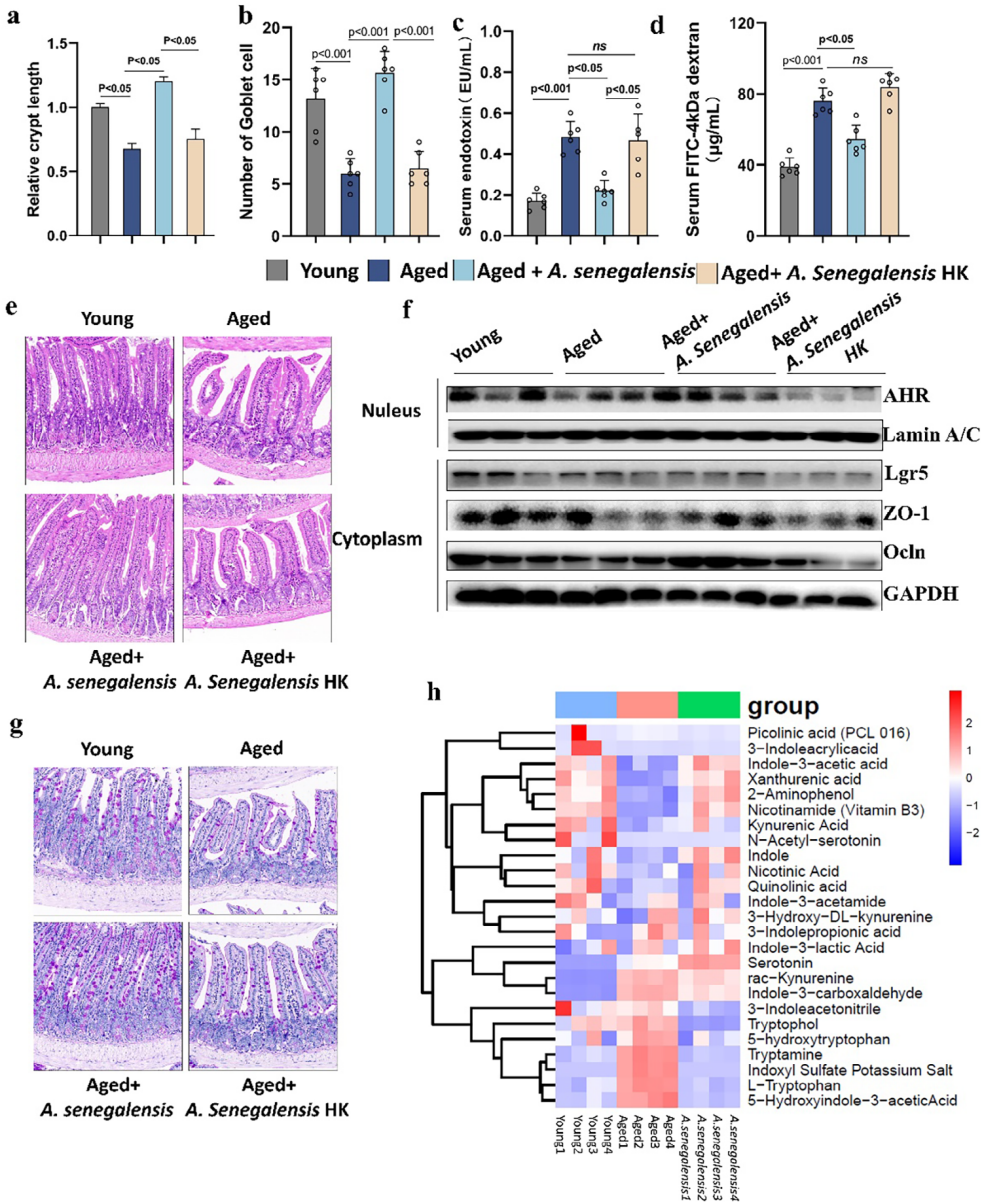
*Alistipes Senegalensis* reduced aging‐induced inflammation and gut barrier function. Young, Aged mice were divided into four groups and treated daily with active and heat‐inactivated *Alistipes Senegalensis* (1 × 10^9^ colony‐forming units) by oral gavage for 8 weeks. Senescence traits including a) Crypt length, b) Number of goblet cell, c) Serum endotoxin, d) FITC‐4 kDa dextran were measured. e) Representative morphology images of H&E staining of small intestine, Scale bar = 100 µm. f) The protein level of AhR, Lgr5, ZO‐1 and Occludin were detected by immunoblot. g) Representative pictures of PAS staining, Scale bar = 100 µm. h) Heatmap of different metabolite abundance associated with tryptophan pathway via targeted metabolome. Data are presented as the mean ± s.e.m.

As shown by previous research, *A. senegalensis* is an indole‐producing species within the mammalian intestinal tract; however, its physiological functions are not well understood.^[^ ^28^ ^]^ Due to its enrichment in GPN‐treated mice and its capacity for indole production, it was hypothesized that *A. senegalensis* plays a pivotal role in gut barrier repair and anti‐inflammaging effects of ginseng polysaccharides. To test this hypothesis, 18‐month‐old mice were treated with *A. senegalensis*. Furthermore, to verify whether the biological activity of *A. senegalensis* is associated with its metabolic pathway, heat‐killed *A. senegalensis* (treated at 95 °C in a water bath for 15 min) was administered to aged mice as a negative control and designated as the *A. senegalensis* HK group (**Figure** **9**).

### 2.7 *Alistipes senegalensis* Alleviated Age‐Related Gut Barrier Impairment, Stemness Attenuation, and Systemic Inflammation through the Indole/AhR Pathway

It was shown that *A. senegalensis* supplementation significantly increased both crypt lengths and the number of goblet cells, together with reduced levels of serum endotoxin and FITC‐dextran, indicating a significant role for *A. senegalensis* in gut barrier repair (Figure 9a–d). As expected, treatment with heat‐killed bacteria counteracted the beneficial effect of *A. senegalensis* treatment. Morphological data are shown in Figure 9e,g, demonstrating that *A. senegalensis*‐treatment markedly improved the structural integrity of the small intestine and elongated the intestinal villi. In addition, the mucosa of the small intestine after *A. senegalensis* treatment was thicker than that in the 18‐month‐old control mice and the connective tissue of the lamina propria was compact. However, the *A. senegalensis* HK treatment resulted in few or no protective effects in aged mice. To dissect the relationship between *A. senegalensis* and the AhR signaling pathway, AhR activation was investigated. AhR activation was found to be inhibited together with the age‐decreased indole level, while the nuclear translocation of AhR was significantly increased in the *A. senegalensis* group. Thus, the changes in gut barrier markers and stem cells corresponded with AhR activation. The expression of AhR, ZO‐1, and Lgr5 was comparable to the aged group (Figure 9f; Figure S5h, Supporting Information).

These results were in agreement with the hypothesis that *A. senegalensis* induced barrier repair through its tryptophan metabolism and indole production. Targeted metabolomics results confirmed this speculation, as shown in Figure 9h, where the excessive accumulation of tryptophan and reduced indole levels in feces suggested that aged mice have reduced tryptophan metabolic capacity, as found previously^[^ ^29^ ^]^ while administration of *A. senegalensis* elevated the levels of tryptophan metabolism and its related metabolites, indicating the pivotal role of *A. senegalensis* in GPN‐induced gut barrier repair.

**Figure 10:**
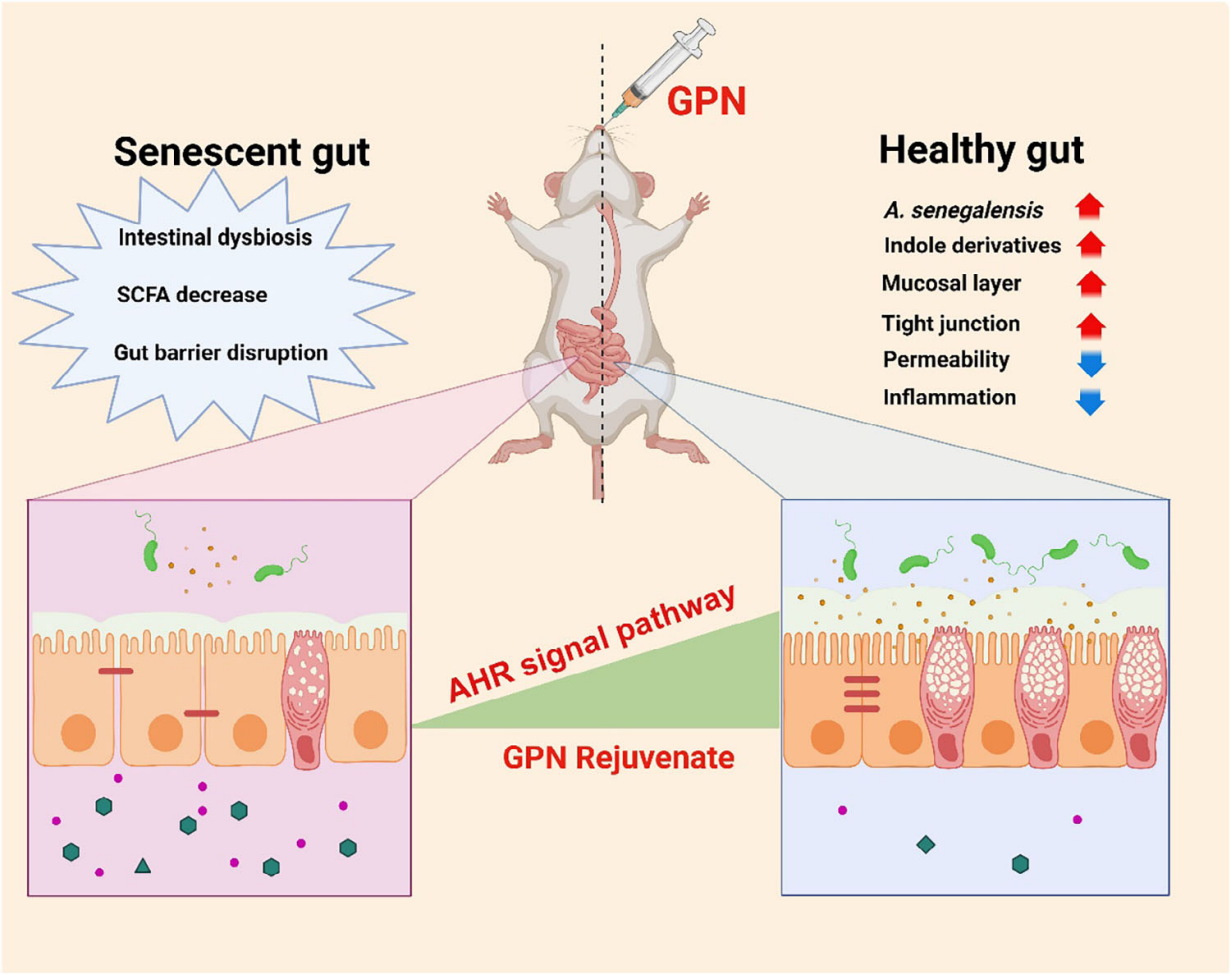
Proposed model for gut barrier repairment effects of GPN in aged mice through the *Alistipes senegalensis*‐indoles‐AhR functional axis.

In summary, the findings identified a bacterium, *A. senegalensis*, that showed high levels of tryptophan metabolism and indole production in GPN‐treated aged mice. *A. senegalensis* prevented age‐related gut barrier dysfunction and inflammaging through increased indole synthesis and AhR activation, which was involved in the beneficial effects of ginseng neutral polysaccharide GPN (**Figure** **10**).

## 3 Discussion

*Panax ginseng* C. A. Meyer, a vital Chinese traditional medicinal plant, displays various biological functions, including immunomodulation and anti‐aging properties. Ginseng‐derived polysaccharides, one of the main active ingredients, represent a novel class of natural prebiotics that are responsible for intestinal metabolism and modulation of the gut microbiota. Our previous research demonstrated that long‐term administration of GPN significantly affected the composition of the gut microbiota and alleviated intestinal inflammation. However, the precise pathway involved in the effects of ginseng polysaccharides has not been clarified. Therefore, further studies are required to elucidate the underlying mechanisms by which ginseng polysaccharides influence bacterial populations and host health. In this study, we identified *A. senegalensis*, a beneficial bacterium that predominated in GPN‐treated mice, and confirmed its ability to protect intestinal homeostasis in aged mice as well as extend the lifespan of *C. elegans* via the AhR pathway. These results provide causative clues for understanding the role of gut microbiota in the beneficial effects associated with GPN.

The human body is a “superorganism” with trillions of commensal bacteria in its gastrointestinal tract. The composition of the microbiota remains relatively stable throughout adulthood, while the microbiota of older adults tends to be enriched in pathobionts and displays a decreased abundance of beneficial commensals. In experimental invertebrate model systems, it has been confirmed that some bacteria and small molecules secreted by these bacteria are associated with host longevity in the context of simple bacterial background.^[^ ^30^, ^31^, ^32^ ^]^ However, little is known of how these are affected by aging and how cell signaling pathways could be targeted to delay the age‐related decline in barrier function. In addition, it is difficult to identify a specific factor involved in accelerating or mitigating aging from a complex gut bacterial community and elucidating their mechanisms in mammals. Despite this, emerging evidence has revealed that a healthy and stable microbiota remains throughout the adult stage and is diversified in an age‐dependent manner.^[^ ^33^, ^34^ ^]^ Indeed, the “aged microbiota” should be examined particularly in terms of enrichment in pathobionts and decreased abundance of bacteria with anti‐inflammatory and immunomodulatory properties, such as the *Bacteroides, Faecalibacterium, Ruminococcus, Alistipes, Coprococcus, Roseburia, Parabacteroides, Coprobacillus, Anaerotruncus*, and *Bifidobacterium* genera.^[^ ^35^, ^36^, ^37^, ^38^, ^39^ ^]^

Among these commensals, *Alistipes*, a relatively new bacterial genus, is an anaerobic microbe found mostly in the healthy human gastrointestinal tract microbiota and is relevant to both dysbiosis and disease.^[^ ^40^, ^41^, ^42^, ^43^ ^]^ In recent years, several studies have investigated the alterations in bacterial abundance for *Alistipes* in relation to disease occurrence. Several reports are consistent with the findings of the present study, showing that *Alistipes* is implicated in inflammatory diseases, including liver fibrosis,^[^ ^32^ ^]^ IBD^[^ ^40^ ^]^ and mood disorders^[^ ^41^ ^]^ due to its unique capacity for amino acid fermentation. However, investigations focusing on the immunological and mechanistic implications by which *Alistipes spp*. are correlated with human health are still rare. In this study, the results showed reduced abundances of *Alistipes senegalensis*, *Alistipes timonensis*, and *Alistipes indistinctus* in aged mice, and the marked enrichment of these, especially *A. senegalensis*, after GPN administration. In addition, it has been reported that *A. senegalensis* and *Alistipes timonensis* contain catalase activity and can hydrolyze tryptophan to indoles. In summary, *A. senegalensis* was found to have significant protective effects on the gut barrier in GPN‐treated aged mice and suggested that indole production leading to activation of the AhR pathway was responsible for this.

Microbiota produce a wide range of metabolites that impact host systemic cellular functions. The FMT and FCM result confirmed that gut microbiota remodeling and changes in bacterial metabolites are indispensable for the beneficial therapeutic effects of GPN. It worth noting that indole is arguably the major regulator of intestinal barrier function and gut stem cell differentiation. Indole and its metabolites, the product of commensal bacterial decomposition of tryptophan,^[^ ^26^ ^]^ have been identified as natural AhR ligands that promote the protective response against acute stressors, including infection and hyperinflammatory immune responses.^[^ ^44^, ^45^ ^]^ Recent research has shown that the contents of indole and tryptophan decrease in an age‐dependent manner, suggesting that the elderly population has microbiota that are unable to generate indoles and metabolize tryptophan or produce these metabolites at very low levels.^[^ ^29^ ^]^ Similar results were found in the targeted metabolomic results, showing that tryptophan hydrolysates and indole contents were reduced in aged mice and recovered to levels comparable to or higher than those in the young group after *A. senegalensis* supplementation. To date, the signatures of indole and its metabolites suggest their potential as appropriate biomarkers of chronological age. The beneficial effects exerted by indoles on aging are mainly attributed to the activation of gut stem cells and repair of the epithelial barrier by acting as AhR ligands. AhR, a transcription factor activated by ligands, is widely expressed and plays a major and beneficial role in the gut microenvironment.^[^ ^46^ ^]^ In addition to its well‐documented function in intestinal immune cells, AhR appears to exert a more important role in the repair of intestinal barrier integrity, especially in terms of tight junctions and regenerative processes. Upon ligand binding, AhR translocates to the nucleus and binds to the AhR nuclear translocation protein (ARNT), subsequently promoting the transcription of a variety of downstream target genes, including genes of the MLCK‐pMLC signaling pathway ^[^ ^47^ ^]^ leading to upregulation of intestinal epithelial tight junction proteins. In addition, indole induces IL‐22 to promote stem cell‐mediated repair of the epithelial barrier via AhR.^[^ ^23^, ^48^ ^]^ The results in Caco‐2 cells and *C. elegans* also confirmed that GPN‐FCM administration significantly promoted AhR nuclear translocation and promoted binding between AHR and ARNT, suggesting that this represents the underlying mechanism by which GPN induces increased expression of intestinal epithelial tight junction proteins and stem cell activity.

It is plausible that gut barrier function and gut microbiota play critical roles in systemic inflammation and healthy aging, which has led to the revival of the “leaky gut” concept.^[^ ^49^, ^50^ ^]^ The results in aged mice demonstrated that the severity of the aging phenotype was associated with reduced gut barrier function. Recent experiments in Drosophila have shown that impaired gut barrier integrity is associated with age‐associated metabolic and inflammatory patterns, and are recognized as a marker of “impending” death. In addition, the use of intestinal organoids and laboratory animals has led to new information that sheds light on the aging process in the gut. Gut barriers are complex but well‐orchestrated with selective permeability that restricts the translocation of antigens, endotoxins, and microbes from the gut to the blood, with increased gut permeability elevating the level of systemic inflammation. Thus, targeting the restoration of intestinal barrier function is a promising therapeutic approach. The intestinal epithelium has an extremely high turnover rate and maintenance of its function is associated with the integrity of the tight junctions and the self‐renewal of gut stem cells. As expected, GPN intervention significantly reversed age‐related damage to the tight junctions and Lgr5‐positive stem cells together with enhancing the physiological function and health of the aged mice. These observations were reinforced by GPN‐FCM treatment of organoids and Caco‐2 and THP‐1 cells that highlighted increased levels of ZO‐1 and Lgr5 but reduced levels of inflammatory markers compared with the Age‐FCM group. The combined analysis identified the *A. senegalensis*‐indole‐AhR pathway, by which GPN exerts anti‐inflammaging and gut‐repair effects. The relevance of GPN and gut barrier function to healthy aging and the results presented in this study will provide opportunities for the development of putative and innovative plant polysaccharide strategies for healthy aging.

## 4 Experimental Section

### Preparation and Analysis of the Chemical Composition of GPN

The ginseng was crushed and soaked in distilled water overnight and decocted in water the next day in a 1:10 ratio. The extracts were combined, filtered, and concentrated. Four volumes of anhydrous ethanol were added to concentrate and allowed to stand overnight at 4 °C. The material was then centrifuged to yield the crude polysaccharide GP. The GP was then dissolved in distilled water and dialyzed (2000 Da cutoff) to remove small molecules. The material was then concentrated and lyophilized. The molecular weight was determined by high‐performance gel permeation chromatography (HPGPC) using high‐performance liquid chromatography with a differential detector. Infrared spectra were determined using potassium bromide with a scanning range of 4000‐400 cm^−1^ with a Tenor 27 spectrophotometer (Shimadzu Corporation, Shanghai, China).^1^H‐NMR spectra were measured at 20 °C using an Avance 600 MHz spectrometer (Bruker, Germany).^[^ ^51^ ^]^

### Mouse Experiments

Two‐ and eighteen‐month‐old BALB/c mice were kept in regular filtertop cages. The mice were fed with a standard chow diet and sterile water and were maintained in a 12‐h light: 12‐h dark cycle. The mice were randomly assigned to three groups, namely, the young group and the aging groups with and without GPN supplementation. GPN (200 mg kg^−1^) or PBS was administered by oral gavage for 8 weeks. For experiments, fresh stool samples were collected and stored at ‐80 °C. Intestinal tissues and contents were collected at the termination of the experiment. All animal protocols were approved and conducted according to the guidelines of the Institutional Animal Care and Use Committee at Changchun University of Chinese Medicine (Approval No. 2023094).

### Fecal Microbiota Transplantation (FMT)

Recipient mice (BALB/c, 2‐months‐old and 18‐months‐old) were divided into four groups as shown in Figure 3A (n = 8 in each group). The mice received oral doses of 100 µL of antibiotic cocktail (1 g L^−1^ neomycin, 1 g L^−1^ ampicillin, 1 g L^−1^ metronidazole, and 0.5 g L^−1^ vancomycin) for 14 consecutive days for gut cleansing. On completion of antibiotic cocktail administration, four doses of polyethylene glycol (425 g L^−1^, 200 µL per mouse) were given by oral gavage at 20 min intervals to deplete 95% of the live microbial counts in the feces.^[^ ^52^, ^53^ ^]^

All gavage samples were freshly prepared. Fresh feces from the donor mice in each group (young, aged with or without GPN) were collected. Fecal samples (0.5 g) were placed in a sterilized beaker, followed by the addition of 5 ml of sterile normal saline containing deoxidizer, with stirring at 37 °C. Each fecal sample (0.5 g) was suspended in 5 mL of reduced sterile PBS (0.05% L‐cystein‐HCL and N_2_), vortexed, and sedimentation 5 min each. The slurry was then filtered through a membrane with 100‐µm pores. The fecal microbial transplant (FMT, 200 µL per mouse) was administered by gavage once a day for 21 days.^[^ ^54^, ^55^, ^56^ ^]^

### Fecal Water Content Assay

Five fresh fecal pellets per mouse were collected into a tube at 08:30 and measured to record the wet weight. Open tubes containing the feces were placed in an oven and dried at 80 °C for 24 h. The weight was then measured and recorded as the dry weight. The fecal water content per mouse was calculated according to the following equation:

(1) $$fecalwatercontent\left(\%\right)=\left(1-\frac{fecaldryweight}{fecalwetweight}\right)\times 100$$

### Histological Analysis

After the euthanasia of the mice, samples of the small intestine were harvested and fixed immediately in ice‐cold 4% paraformaldehyde, followed by embedding in paraffin and cutting into 5 µm sections. Hematoxylin and eosin (H&E) staining was conducted to assess morphological changes. For goblet cell staining, PAS staining was performed on deparaffinized and rehydrated sections. The quantification of goblet cells was expressed as the total number of PAS‐positive cells in each intestinal crypt and the total number of goblet cells was counted from four different fields. The lengths of the intestinal crypts were determined using ImageJ (NIH, Bethesda, MD, USA). The “Analyze‐Set Scale” and the straight‐line tool were used to set measuring scales and the intestinal crypt lengths, respectively. Eight fields per section were selected randomly, with average values used for analysis.

### Gut Permeability and *Endotoxin* Assays in Mice

Gut permeability was assessed using FITC‐dextran assays. Briefly, 1 mg g^−1^ body weight of FITC‐dextran (4 kDa Sigma, St Louis, MO, USA) was administed orally to mice after 4 h of fasting. Blood samples were collected after 4 h and centrifuged (10 000 x *g* at 4 °C) for 10 min. The sera were placed in 96‐well microtiter plates and the FITC fluorescence was measured at 485 nm excitation and 530 nm emission wavelengths using with an Infinite M200 plate reader (Tecan, Mannendorf, Switzerland), with quantification against serially dilutions of the FITC‐dextran marker as the standard curve.

Markers of systemic gut permeability, including LPS‐binding protein (LBP, Hycult Biotech, PA, USA) and soluble cluster of differentiation 14 (sCD14, R&D Systems, MN, USA) were detected in serum using ELISA kits. Endotoxins in the systemic circulation were measured using the Pierce Chromogenic Endotoxin Quantitation Kit (Pierce Biotechnology, IL, USA), according to the manufacturer's instructions.

### Fecal Conditioned Medium (FCM) Preparation

The fresh feces snap‐frozen in liquid nitrogen were crushed using a mortar and pestle. The resultant powdered feces (100 mg) were suspended in cold DMEM (Gibco, Waltham, MA, USA) at 100 mg feces in 100 mL of medium and placed on a shaker (200 rpm) in the cold room for 1 h to allow proper mixing. The suspension was then filtered through 0.45 and 0.22 µm filters in succession under sterile conditions. The FCM was used at 1:40 dilutions for treating cells and intestinal enteroids^[^ ^49^, ^57^ ^]^ while 1:10 dilutions of FCM were used for *C. elegans*.

### Co‐Culture Transwell System

Caco‐2 cells (HTB‐37, ATCC) were seeded into the upper compartment of 12‐well Transwell plates (polycarbonate membrane filters, pore size 3.0 µm) at a density of 2 × 10^4^. THP‐1 cells were co‐cultured in the lower compartment with Caco‐2 cells. The detailed methods were described previously.^[^ ^41^ ^]^ When the Caco‐2 cells reached 70–80% confluence, they were treated with different FCMs, specifically, from the young, aged with or without GPN administration groups for 48 h. Both the Caco‐2 and THP‐1 cells were then collected for experiments.

### Enteroid Development and Treatments

Complete small intestines were harvested from 2‐month‐old mice and were rinsed with pre‐cooled Duchenne PBS (DPBS, Lonza, MD, USA). The ilea were cut longitudinally, rinsed with chilled PBS, sliced into pieces (2 mm), and placed in 50‐mL tubes in chilled PBS. After incubation for 15 min with shaking in 25 mL of pre‐warmed trypsin (Gibco), the tissues were washed with 10 mL pre‐cooled PBS with 0.1% BSA (Thermo Fisher, Waltham, MA, USA), followed by filtration (70‐µm cell strainer) and centrifugation (290 × g, 5 min, 4 °C). The precipitate containing the crypts was added to 10 mL of cold DMEM/F‐12 (Gibco), followed by recentrifugation (500 × g, 10 min) and resuspension in 150 µL of IntestiCult Organoid Growth Medium (Stem Cell Technology, Canada) with gentamicin (50 µg/mL, Gibco). This was followed by the addition of Matrigel (Thermo Fisher), after which a dome was formed with 50 µL of the material in the center of wells of pre‐heated 24‐well plates and incubated (30 min, 37 °C with 5% CO_2_) until the Matrigel had solidified. The organoid growth medium was replaced three times per week until day 10 when FCM was used to treat the organoids (3 replicates/group) followed by harvesting after two days and assessment of protein levels and crypt diameters.^[^ ^57^ ^]^

### *C. elegans* Culture and Longevity Assay

Wild‐type N2 worms, mutant strain CZ2485, and transgenic strains SJ4143 and UL1709 were purchased from the Caenorhabditis Genetics Center (CGC, University of Minnesota, MN, USA). Lifespan determination and locomotion assays were performed as described in the previous research.^[^ ^58^ ^]^

### Measurement of Gut Permeability in *C. elegans* Using Smurf Assay

The trial was conducted as previously described.^[^ ^41^ ^]^ Briefly, animals were raised for lifespan determination as described above and compared after treatment with different FCMs. The animals were suspended in standard OP50 bacterial liquid cultures mixed with blue food dye (Spectrum FD&C Blue #1 PD110, 5.0% w/v in water) (Spectrum Chemical) for 3 h. The animals were then placed on unseeded NGM plates. When the NGM plates were sufficiently dry to collect the worms, the worms were transferred to glass slides and fixed with 2% agarose gel, and the presence of blue food dye in the body cavity was assessed and imaged using an Eclipse TE300 microscope (Nikon, Japan) at 4x magnification. Three or more independent experiments were performed at each time point, with 10–15 animals per treatment.

### Untargeted Metabolomics Analysis

After metabolite extraction from the different groups of cecal contents, off‐target metabolomics analysis was performed in 900 µL of acetonitrile: methanol: H_2_O = 4:4:2. UHPLC‐MS/MS was conducted by Personal Biotechnology Co., Ltd (Shanghai, China) using an Agilent 1290 Infinity LC system (Agilent Technologies, Santa Clara, CA, USA) with a Triple TOF 6600 mass spectrometer (AB SCIEX) using both positive and negative ion modes. ProteoWizard software was utilized to convert the MS data to mzXML format, while an in‐house R package was applied for additional processing of the raw data for detecting, extracting, aligning, and integrating the peaks. Annotation of the metabolites was performed using the Human Metabolome Database (HMDB) and the BiotreeDB V2.1 secondary MS database using 0.3 as cutoff. QC analysis was used to assess the reliability and suitability of the system for further analysis through evalution of the quality of the samples and the methods and stability of the system. Overall metabolic changes were compared between the three groups using PCA and OPLS‐DA. Model reliability was assessed using the criteria of R2Y and Q2 > 0.05 and variable importance in projection (VIP) values. Differentially expressed metabolites (DEMs) were identified utilizing the criteria of p < 0.05, VIP >1, |fold change| > 1.0. Pathways showing significant enrichment (p < 0.05 and effects >0.1 were identified and “ggplot2” and “pheatmap” in R were utilized respectively to generate volcano plots and heatmaps.

### Gut Microbiota Analysis

Cecal contents from each group of mice (n = 8 per group) were frozen immediately in liquid nitrogen and kept at ‐80 °C. Macrogenomic sequencing was conducted by Personal Biotechnology Co., Ltd. Extraction of genomic DNA was performed with an OMEGA Mag‐Bind Soil DNA Kit (M5635‐02), following the provided directions, followed by storage at ‐20 °C. The DNA was evaluated quantitatively using a Qubit™ 4 fluorometer (Invitrogen, Waltham, MA, USA) and Qubit™ 1X dsDNA HS Assay Kit (Q33231) and qualitatively with agarose gel electrophoresis. Metagenomic shotgun sequencing libraries (insert size, 400 bp) were prepared with an Illumina TruSeq Nano DNA LT Library Preparation Kit (Illumina, San Diego, CA, USA), with sequencing on an Illumina NovaSeq platform with sequencing strategy PE150 (Shanghai, China).

Quality reads were acquired from the raw data by quality control and host filtration and were analyzed using Kraken2 on a RefSeq‐based database including archaea, bacteria, viruses, fungi, protozoa, metazoans, and viruses. Clustering of CDS regions was performed mmseqs2 in the “easy clustering” mode, utilizing identity thresholds of 0.90 for protein and 0.90 coverage for shorter alleles. Gene abundance was assessed by mapping the high‐quality reads to predicted sequences with the Salmon quasi‐mapping model with a ′–meta–minScoreFraction = 0.55″ and CPM (per million mapped reads/thousand base copies) for normalization of abundance in the metagenome.

### *Alistipes enegalensis* Culture and Treatment

*A. senegalensis* was obtained from BeNa Culture Collection (BNCC) and was cultured on brain heart infusion (BHI) agar. Plate with an anaerobic gas mixture (80% N_2_, 10% CO_2,_ and 10% H_2_) at 37 °C. The cultures were centrifuged at 2500 × g for 5 min at 4 °C, washed twice with sterile anaerobic PBS, and resuspended for a final concentration of 1 × 10^9^ CFU/200 µl under strictly anaerobic conditions. Each mouse was gavaged daily with 200 µL of sterile PBS or pretreated *A. senegalensis* for 8 weeks.^[^ ^59^ ^]^

### Plate Counting

The same volumes of *A. senegalensis* suspensions were spread on BHI agar plates with GPN (1 and 2 g mL^−1^) or without GPN and grown for 120 h. The bacteria were collected, homogenized with 200 mL PBS, and centrifuged. One milliliter of the supernatant containing the bacteria was diluted with PBS at a ratio of 1:10000, spread on BHI agar, and incubated anaerobically at 37 °C for 120 h. The colonies were counted and the concentrations of the bacterial suspensions collected from the blank and GPN‐treated plates were calculated.

### Metabolic Profiling Analysis

The LC‐MS/MS analysis of the samples was conducted on a Nexera Series LC‐40 system coupled with a QTRAP® 6500+ mass spectrometer (Sciex, USA) at Majorbio Bio‐Pharm Technology Co. Ltd. (Shanghai, China). The samples were separated using an ACQUITY UPLC® HSS T3 column (2.1 × 150 mm, 1.8 µm) at a constant temperature of 40 °C. The metabolites were separated using a 1 mL min^−1^ flow rate with a gradient consisting of the mobile phases of 0.1% formic acid in water (solvent A) and 100% acetonitrile in water containing 0.1% formic acid (solvent B). The total chromatographic separation duration was 18 min.

The MS data were collected on a UHPLC system coupled to a QTRAP® 6500+ mass spectrometer (Sciex, USA) equipped with an electrospray ionization (ESI) source operating in both positive and negative modes. The raw LC‐MS data were converted to the Sciex software OS. All ion fragments were automatically identified and integrated by using default parameters, and all integration was checked manually. The metabolite concentrations of the samples were calculated using linear regression standard curves.

### Statistical Analyses

Data were analyzed using GraphPad Prism 8.2 (GraphPad, San Diego, CA, USA). Differences between two groups were assessed using unpaired two‐tailed Student`s t‐tests. One‐way analysis of variance (ANOVA) with Bonferroni`s post hoc test was used for comparisons among multiple groups. The life cycle experiments were analyzed using log‐rank (Mantel‐Cox) analysis. Unless otherwise stated, the threshold for statistical significance was set at *p* < 0.05 to determine differences between groups for cytokine analysis, intestinal permeability, metabolites, gene expression, and microbiota.

### Ethics Approval

This study was approved by Institutional Animal Care and Use Committee at Changchun University of Chinese Medicine (Approval No.: 2023094). Experiments were performed in accordance with the guidelines.

## Conflict of Interest

The authors declare no conflict of interest.

## Author Contributions

D.W. and H.W. contributed equally to this work. D.‐D.W. and H.W. performed the experiments and interpreted the results. D.‐D.W., L.‐W.S., and S.Z. conceived the project and designed the research. Y.‐N.L. and F.‐B.L. provided the GPN extract and polysaccharides fractions. J.L. and X.‐L.T. contributed essential animal experiments. D.‐D.W., H.W., and Y‐N.L. wrote the manuscript. D.Y. and D‐Q.Z. provided essential reagents and tools. all authors discussed the results and approved the manuscript.

## Supporting information



Supporting Information

## Data Availability

Data are available in a public, open access repository. The Macrogenomic sequencing data presented in the study were deposited in the NCBI Sequence Read Archive repository, accession number SRP345072. And Untargeted metabolomics data presented in the study were deposited in the MetaboLights database, accession number MTBLS11662.
