# Supplementary material for: Alistipes senegalensis is Critically Involved in Gut Barrier Repair Mediated by Panax Ginseng Neutral Polysaccharides in Aged Mice
Source: Adv Sci (Weinh). 2025 Jul 3;12(36):e16427. doi: 10.1002/advs.202416427 (PMC12462932; doi:10.1002/advs.202416427)
Supplement: Supplementary file 1 — Supporting Information [file ADVS-12-e16427-s001.docx]

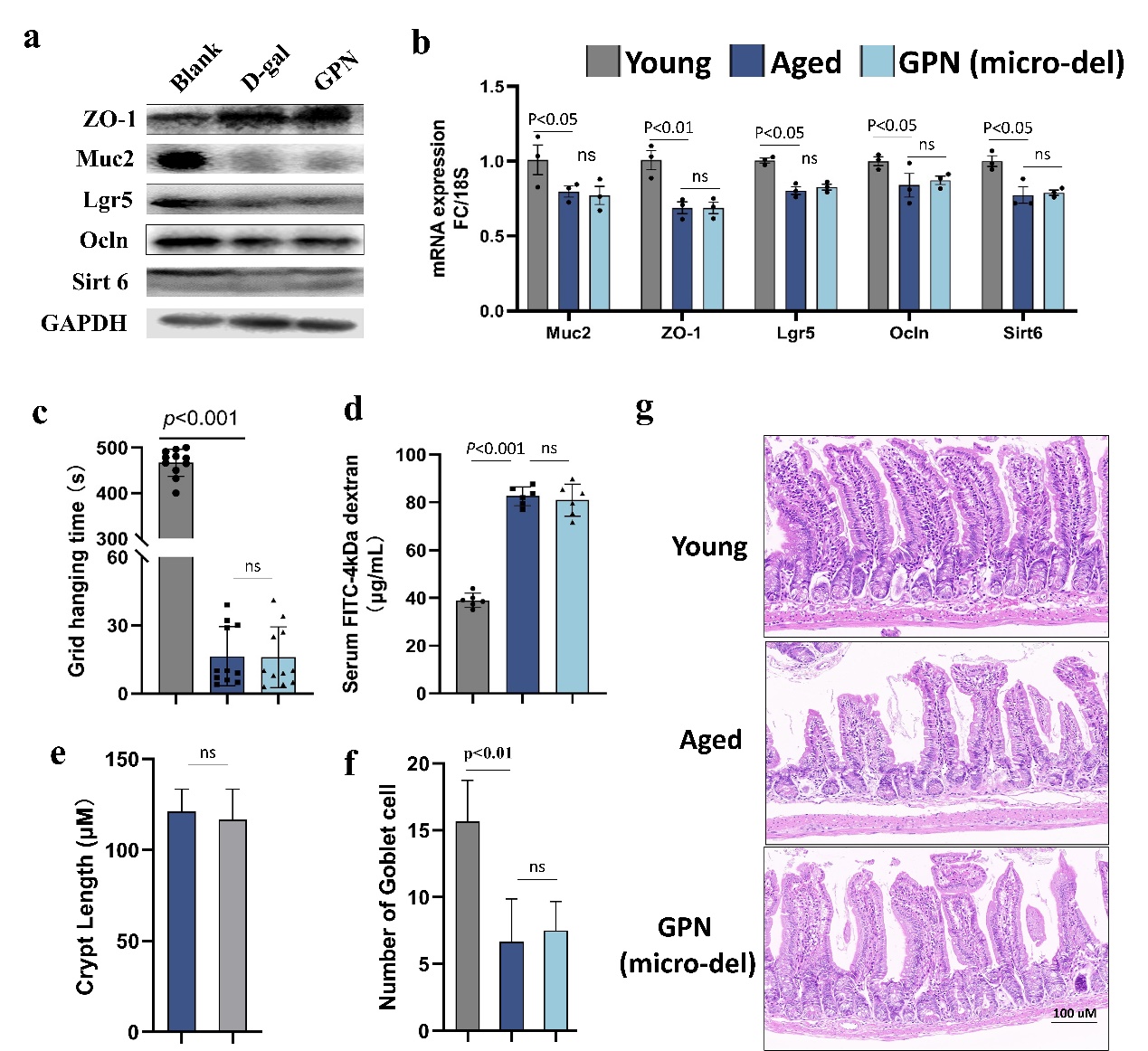


**Figure S1 The anti-aging effects of GPN intervention disappears in the condition of gut microbiota lackness. (a)** Western blot results and **(b)** mRNA expression of ZO-1, Ocln, Muc2, Lgr5 and Sirt6 in Caco-2 cells treated with GPN (100μg/mL) with or without D-gal (100μM). Microbiota deleted aged mice were treated with GPN, **(c)** Grip hanging time, **(d)** Intestinal permeability, **(e)** Crypt length, **(f)** Number of the goblet cell and **(g)** H&E staining of small intestinal. Data are presented as the mean ± s.e.m.


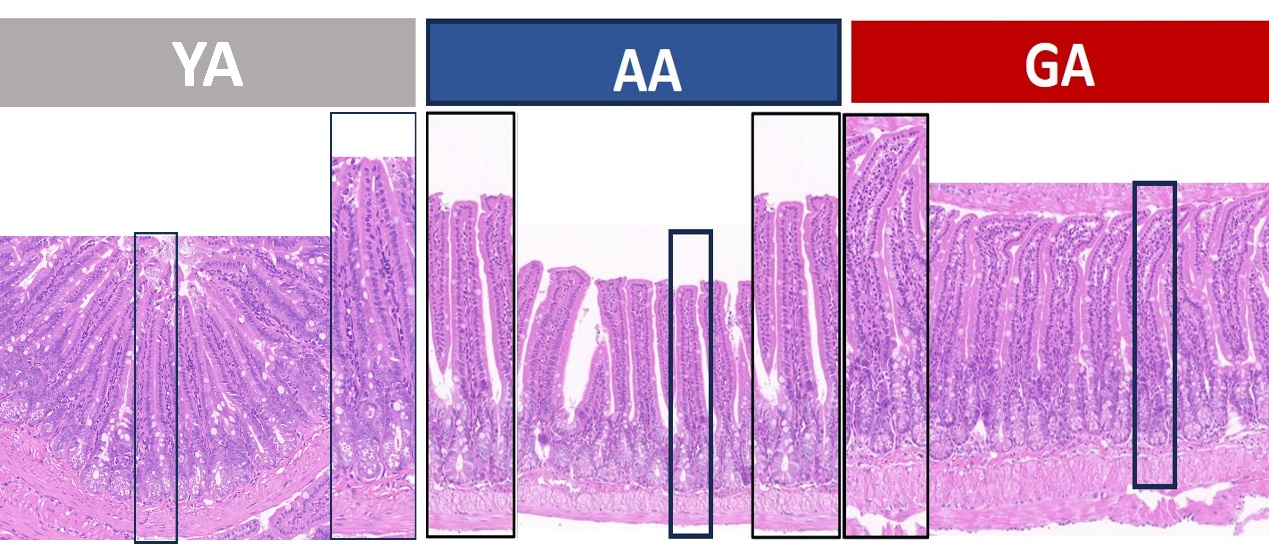


**Figure S2 Visualized images of H&E staining of GA, AA and YA group.**


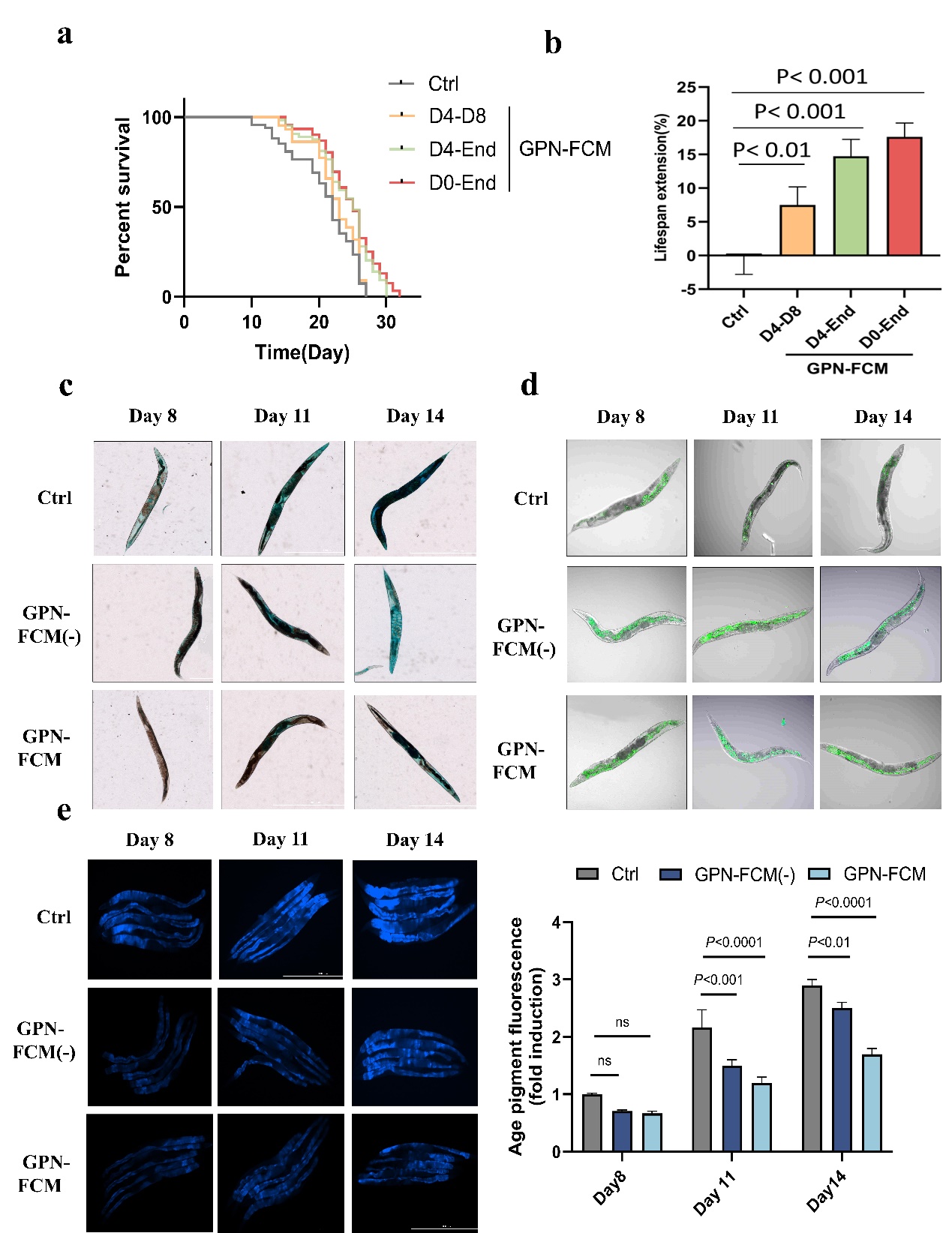


**Figure S3 Long-term effects evaluation of GPN enhance beneficial metabolites on intestinal barrier repairment. (a)** Survival curves and **(b)** Percentage changes of the lifespan of N2 worms treated with GPN-FCM for different timing stages (N=75 for each group). **(c)** Smurf assay images of N2 worms, **(d)** Representative images of ges-1::GFP (mit) in SJ4143 worms and **(e)** Fluorescence images and quantitation of lipofuscin accumulation with N2 nematodes on day 8,day 11 and day 14. GPN-FCM (-): treated with GPN-FCM from day 4 to day 8; GPN-FCM: treated with GPN-FCM for whole lifespan, n=15 for each group. Scale bar = 100μm. Data are presented as the mean ± s.e.m.


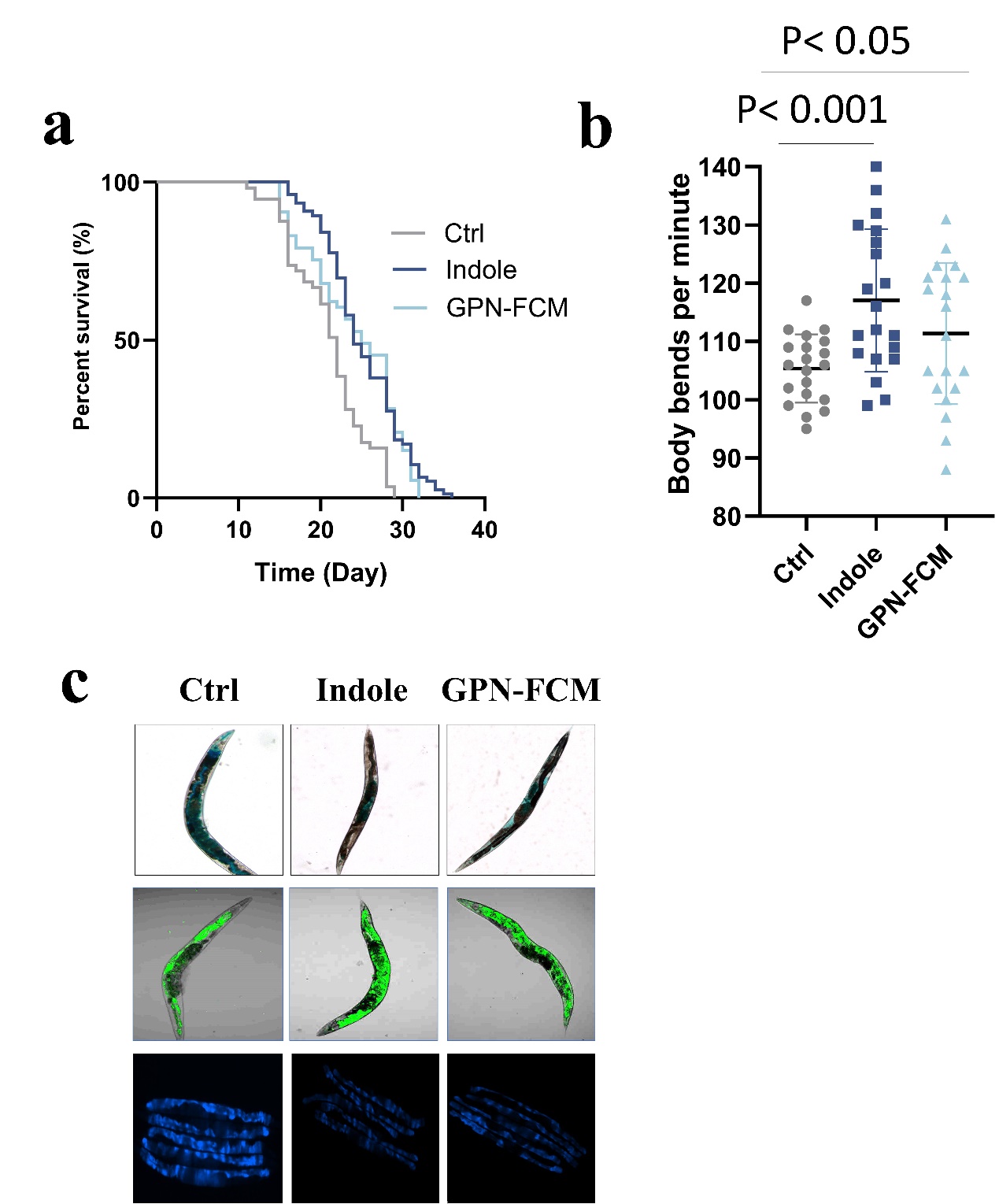


**Figure S4 The alleviation of gut leaky and the extension of lifespan by indole. (a)** Survival curves and **（b）**Locomotor performances of N2 worms (N=80 for each group). **(c)** DIC images of N2 animals after soaking in blue food dye, representative images of ges-1::GFP (mit) in SJ4143 worms and autofluorescence of lipofuscin accumulation in N2 nematodes, n=15 for each condition. Worms of N2 and SJ4143 strains were fed with indole (250 μm) and GPN-FCM for whole lifespan, images were obtained on day 13, scale bar = 100 μm. Data are presented as the mean ± s.e.m.


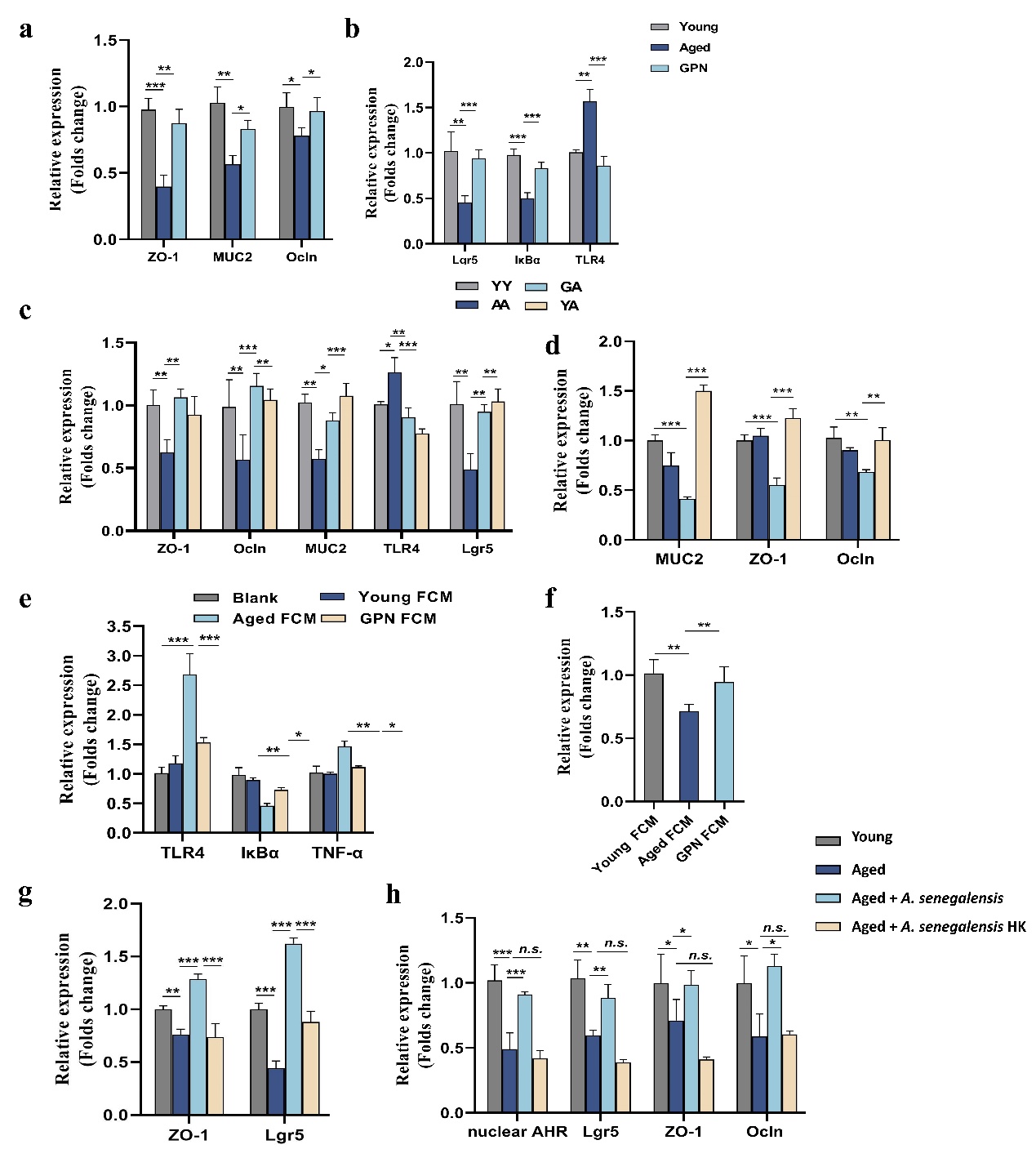


**Figure.S5 The expressions of proteins were quantified by the ratio of total amount protein/GAPDH or Lamin A/C.** All data shown are representative of 3 independent experiments. **(a, b)** Quantification of proteins expressions for Fig. 2 g and l. **(c)** Quantification of proteins expressions for Fig. 3h. **(d, e)** Quantification of proteins expressions for Fig. 4e. **(f)** Nuclear AHR quantified by the ratio of AHR/Lamin A/C in Fig. 6a. **(g)** Quantification of proteins expressions for Fig. 6a. (h) Quantification of proteins expressions for Fig. 8f. Bars in graphs represent mean ± SD. **p*<0.05, ***p*<0.01, ****p*<0.001.
